# Predicting the molecular subtypes of breast cancer using nomograms based on three-dimensional ultrasonography characteristics

**DOI:** 10.3389/fonc.2022.838787

**Published:** 2022-08-19

**Authors:** Xiaojing Xu, Liren Lu, Luoxi Zhu, Yanjuan Tan, Lifang Yu, Lingyun Bao

**Affiliations:** Department of Ultrasound, Affiliated Hangzhou First People’s Hospital, Zhejiang University School of Medicine, Hangzhou, China

**Keywords:** breast cancer, molecular subtypes, ultrasonography, relevance of three-dimensional ultrasonography, nomogram

## Abstract

**Background:**

Molecular subtyping of breast cancer is commonly doneforindividualzed cancer management because it may determines prognosis and treatment. Therefore, preoperativelyidentifying different molecular subtypes of breast cancery can be significant in clinical practice.Thisretrospective study aimed to investigate characteristic three-dimensional ultrasonographic imaging parameters of breast cancer that are associated with the molecular subtypes and establish nomograms to predict the molecular subtypes of breast cancers.

**Methods:**

A total of 309 patients diagnosed with breast cancer between January 2017and December 2019 were enrolled. Sonographic features were compared between the different molecular subtypes. A multinomial logistic regression model was developed, and nomograms were constructed based on this model.

**Results:**

The performance of the nomograms was evaluated in terms of discrimination and calibration.Variables such as maximum diameter, irregular shape, non-parallel growth, heterogeneous internal echo, enhanced posterior echo, lymph node metastasis, retraction phenomenon, calcification, and elasticity score were entered into the multinomial model.Three nomograms were constructed to visualize the final model. The probabilities of the different molecular subtypes could be calculated based on these nomograms. Based on the receiver operating characteristic curves of the model, the macro-and micro-areaunder the curve (AUC) were0.744, and 0.787. The AUC was 0.759, 0.683, 0.747 and 0.785 for luminal A(LA), luminal B(LB), human epidermal growth factor receptor 2-positive(HER2), and triple-negative(TN), respectively.The nomograms for the LA, HER2, and TN subtypes provided good calibration.

**Conclusions:**

Sonographic features such as calcification and posterior acoustic features were significantly associated with the molecular subtype of breast cancer. The presence of the retraction phenomenon was the most important predictor for the LA subtype. Nomograms to predict the molecular subtype were established, and the calibration curves and receiver operating characteristic curves proved that the models had good performance.

## Introduction

Breast cancer is a heterogeneous and complex disease. The main molecular subtypes of breast cancer are luminal A (LA), luminal B (LB), human epidermal growth factor receptor2-positive (HER2), and triple-negative (TN) (1, 2).This heterogeneity leads to vast differences in disease progression, treatment response, and prognosis. Therefore, preoperatively identifying different molecular subtypes of breast cancer can be significant in clinical practice. Nomograms have been used extensively for visualizing predictive models in cancer. They present user-friendly graphic presentation of the estimated probabilities of the molecular subtypes. Moreover, nomograms may guide clinical diagnosis and treatment and can help facilitate precision medicine (3).

Hand-held ultrasonography (HHUS) has some limitations because of the lack of standardization (4). Meanwhile, three-dimensional ultrasonography (3D-US) has been essential as a preoperative tool because of its reproducibility and reduced operator dependence. Furthermore, its unique coronal plane can provide additional diagnostic information and potentially improve the characterization of breast lesions. Moreover, the availability of automated breast US in clinical practice is increasing (5, 6). Determining whether preoperative 3D-US can distinguish tumour subtypes has important clinical significance. Previous studies have shown that the characteristics of 3D-US correlated with molecular classification, for example, calcification is associated with the HER2-positive subtype, while the retraction phenomenon is more related to the LA subtype (7–9).

In recent years, artificial intelligence for 3D-US has been applied in the differential diagnosis of benign and malignant breast masses, however there are only a studies on 3D–US in molecular typing (10).

The study aimed to investigate the relevance of the 3D–US imaging characteristics of breast cancer associated with specific molecular subtypes in order to establish nomograms that distinguish the molecular subtypes of breast cancer. This study explored the possibility of predicting molecular typing models with large samples and provided insights into the future artificial intelligence prediction of molecular typing.

## Methods

### Patients

The Institutional Review Board authorized this retrospective study, The requirement for informed consent was waived due to the retrospective nature of the study. Between January 2017and December 2019, 326 patients were consecutively enrolled from our hospital with random selection. All patients had invasive breast cancer, which was histologically diagnosed. Their molecular subtypes were estimated from the surgical specimens. Patients with lesions of undetermined immunohistochemical results (n=12) and patients with considerable deformity of the breast or chest (n=5) were excluded. After these exclusions, 309 patients were enrolled in this study.

### Data collection

Patient information and lesion size and location were recorded. All imaging features were retrospectively reviewed according to the 5th edition of the American College of Radiology Breast Imaging Reporting and Data System lexicon (11). Two radiologists who were blinded to the patients’ previous imaging data and clinical information, age, tumor position, and maximum tumor diameter (according to the TNM[tumor, node, metastasis] stage, the maximum diameter stratifications were(<2cm,2–5cm,and >5cm)reviewed the volume data on an automated breast volume scanner (ABVS)workstation (Siemens Medical Solutions, Mountain View, CA,USA). Analysis of mass lesions included shape (regular or irregular), lesion type (mass or no-mass [no-mass refers to a hypoechoic area lacking a conspicuous margin or shape and can be defined as a non space-occupying lesion]) (12), margin (circumscribed or non-circumscribed), non-parallel/parallel growth, echogenicity (hypoechoic, isoechoic, hyperechoic, or complex), post-acoustic features (enhancement, shadowing, mixed, or no change), calcification types, and axillary lymph node metastasis (round shape, or irregular shape, cortical thickening, asymmetric cortical thickness ≥3 mm, hilar compression, or displacement).

Coronal features included the retraction phenomenon and the skipping sign. The retraction phenomenon was defined as the convergence tendency of the tissue surrounding a lesion with or without cord-like hyperechogenicity intervals on the coronal plane. The skipping sign was defined as anechoic lines around the lesion (13). Elasticity scores were recorded using strain elastography(Tsukuba score, 5 points) (14).

Molecular subtypes were defined according to the 2015revised St. Gallen International Expert Consensus Recommendation (15). Immunohistochemical staining was performed to examine estrogen receptor (ER), progesterone receptor (PR), HER2, and Ki-67 expression. Molecular subtypes were diagnosed according to their hormone receptor and HER2 status, as follows: LA: ER+, PR+, HER2-, and low Ki-67 index; LB: ER+, PR+ or PR-, HER2- or HER2+, and high Ki-67 index; HER2: ER-, PR-, andHER2+; and TN: ER-, PR-, and HER2-. The Ki-67 index was classified as high when≥14% of the tumor cells were immunostained (16).

### Statistical analysis

R software version 4.0.2 (R Foundation for Statistical Computing, Vienna, Austria) was used for all analyses. Statistical significance was set at p<0.05. Nomograms were developed in four steps.First,single factors were compared between the different molecular types using the χ^2^test (parametric) or Fisher’s exact test (non-parametric). The factors of the entire dataset were compared between groups, and the *p* values were calculated. Second, factors with *p*<0.05 were included in the establishment of a four-category prediction model, and a multi-classification regression model was established using multinomial logistic regression for the entire dataset. Third, nomograms were constructed based on the results of the multivariate logistic regression model (17). The total scores of each patient were calculated based on the nomograms, and the probabilities of the LB, HER2, and TN molecular subtypes, with the LA subtype as a reference, were calculated based on these nomograms. Fourth, the performance of the nomograms was evaluated in terms of discrimination and calibration. Calibration curves were used to observe the consistency between the predicted and the true values. The predictive performance of the nomograms was measured using the concordance index and calibration with 1000 bootstrap samples was performed to decrease the overfit bias.

## Results

### General characteristics of the study population

The average age of the study population was 54.25 ± 11.18 years, ranging from 25 to 85 years. There were 288 cases of invasive ductal carcinoma and 21 cases of special types of invasive breast cancer. The general characteristics of the study population, including height, weight, type II diabetes status, and hypertension, are shown in **Table 1**. LB was the most common of the four molecular subtypes (n=137,44.3%), and LA was the second most common (n = 82, 26.6%). The HER2 and TN subtypes were less common(n=40, 12.9% and n=50, 16.2%, respectively).

**Table 1:** General characteristics of patients with breast cancer.

Variables	Patients with breast cancer (n=309)
Age (years, mean ± SD)	54.25 ± 11.18
Height (cm, mean ± SD)	159.08 ± 4.33
Weight (kg, mean ± SD)	60.25 ± 8.78
Sex	
Female	309 (100.0)
Type2 diabetes	
no	286 (92.6)
yes	23 (7.4)
Hypertension	
no	227 (73.5)
yes	82 (26.5)
Mastectomy	
total	263 (85.1)
partial	46 (14.9)
Tumor type	
IDC	288 (93.2)
others	21 (6.8)
Histological grade	
I	21 (6.8)
II	135(43.7)
III	132(42.7)
Molecular subtype	
LA	82 (26.5)
LB	137 (44.3)
HER2	40 (12.9)
TN	50 (16.2)

SD, standard deviation; IDC, invasive ductal carcinoma; LA, luminal A; LB, luminal B; HER2, human epidermal growth factor receptor 2; TN, triple-negative.

### Differences in clinicopathological characteristics,3D-US features, and coronal features among the molecular subtypes

There was no significant difference in age and maximum diameter among the four subtypes. However, the maximum diameter stratifications (<2cm,2–5cm,and >5cm) showed statistical significance. On-mass lesions cannot be expressed in terms of mass description, therefore ‘NA(no answer)’is entered in the table. The detailed distributions of the histologic types are shown in **Table 2**.

**Table 2:** Clinicopathological characteristics of patients with different molecular subtypes.

Variables	Molecular subtype				*p*	test
	LA (n=82)	LB(n=137)	HER2 (n=40)	TN (n=50)		
Age (years, mean ± SD)	55.65 ± 11.00	54.37 ± 11.27	52.80 ± 9.68	52.78 ± 12.30	0.420	ANOVA
Age					0.398	χ2
<60years	51 (62.2)	90 (65.7)	28 (70.0)	38 (76.0)		
≥60years	31 (37.8)	47 (34.3)	12 (30.0)	12 (24.0)		
Maximum diameter (cm, mean ± SD)	2.11 ± 1.27	2.95 ± 1.92	3.02 ± 1.30	2.91 ± 1.94	0.002	ANOVA
Maximum diameter stratification					<0.001	Fisher
<2cm	47 (57.3)	40 (29.2)	8 (20.0)	15 (30.0)		
2-5cm	31 (37.8)	85 (62.0)	28 (70.0)	30 (60.0)		
>5cm	4 (4.9)	12 (8.8)	4 (10.0)	5 (10.0)		
Lesion type					0.418	χ2
no-mass	10 (12.2)	26 (20.4)	7 (17.5)	7 (14.0)		
mass	72 (87.8)	109 (79.6)	33 (82.5)	43 (86.0)		
Location					0.640	χ2
left	42 (51.2)	64 (46.7)	23 (57.5)	25 (50.0)		
right	40 (48.8)	73 (53.3)	17 (42.5)	25 (50.0)		
Irregular mass					0.068	Fisher
no	71 (86.6)	104 (75.9)	32 (80.0)	36 (72.0)		
yes	1 (1.2)	7 (5.1)	1 (2.5)	7 (14.0)		
NA	10 (12.2)	26 (19.0)	7 (17.5)	7 (14.0)		
Non-circumscribed margin					0.123	Fisher
no	72 (87.8)	106 (77.4)	33 (82.5)	39 (78.0)		
yes	0 (0.0)	5 (3.6)	0 (0.0)	4 (8.0)		
NA	10 (12.2)	26 (19.0)	7 (17.5)	7 (14.0)		
Non-parallel mass					0.064	χ2
no	49 (59.8)	84 (61.3)	26 (65.0)	40 (80.0)		
yes	23 (28.0)	27 (19.7)	7 (17.5)	3 (6.0)		
NA	10 (12.2)	26 (19.0)	7 (17.5)	7 (14.0)		
Echogenicity					0.004	exact
complex	4 (4.9)	6 (4.4)	1 (2.5)	8 (16.0)		
hyperechoic	2 (2.4)	0 (0.0)	0 (0.0)	0 (0.0)		
isoechoic	0 (0.0)	0 (0.0)	1 (2.5)	0 (0.0)		
heterogeneous	20 (24.4)	48 (35.0)	17 (42.5)	10 (20.0)		
hypoechoic	46 (56.1)	57 (41.6)	14 (35.0)	25 (50.0)		
NA	10 (12.2)	26 (19.0)	7 (17.5)	7 (14.0)		
Post-acoustic features					0.016	χ2
enhance	14 (17.1)	26 (19.0)	15 (37.5)	18 (36.0)		
shadowing	27 (32.9)	31 (22.6)	8 (20.0)	6 (12.0)		
mix	2 (2.4)	6 (4.4)	3 (7.5)	5 (10.0)		
no-change	29 (35.4)	48 (35.0)	7 (17.5)	14 (28.0)		
NA	10 (12.2)	26 (19.0)	7 (17.5)	7 (14.0)		
Calcification					<0.001	χ2
no	52 (63.4)	50 (36.5)	10 (25.0)	29 (58.0)		
yes	30 (36.6)	87 (63.5)	30 (75.0)	21 (42.0)		
Lymph node metastasis					0.029	χ2
no	59 (72.0)	75 (54.7)	23 (57.5)	36 (72.0)		
yes	23 (28.0)	62 (45.3)	17 (42.5)	14 (28.0)		
Retraction phenomenon					0.001	χ2
no	34 (41.5)	72 (52.6)	27 (67.5)	37 (74.0)		
yes	48 (58.5)	65 (47.4)	13 (32.5)	13 (26.0)		
Skipping sign					0.682	χ2
no	56 (68.3)	87 (63.5)	24 (60.0)	35 (70.0)		
yes	26 (31.7)	50 (36.5)	16 (40.0)	15 (30.0)		
Elasticity score					0.023	χ2
3	11 (13.4)	18 (13.1)	3 (7.5)	10(20.0)		
4	42 (51.2)	64 (46.7)	22 (55)	34 (68.0)		
5	29 (35.4)	55 (40.2)	15 (37.5)	6 (12.0)		

NA (not available) represents missing values.

LA, luminal A; LB, luminal B; HER2, human epidermal growth factor receptor 2; TN, triple-negative; SD, standard deviation; ANOVA, analysis of variance.

### Multifactor multiclass logistic regression analysis

Variables with *p*< 0.05 in the single-factor analysis were entered into the model. These included maximum diameter, irregular shape, non-parallel growth, heterogeneous internal echo, enhanced posterior echo, lymph node metastasis, retraction phenomenon, calcification, and elasticity score.

### Construction of three nomograms to visualize the final model

**Figure 1:**
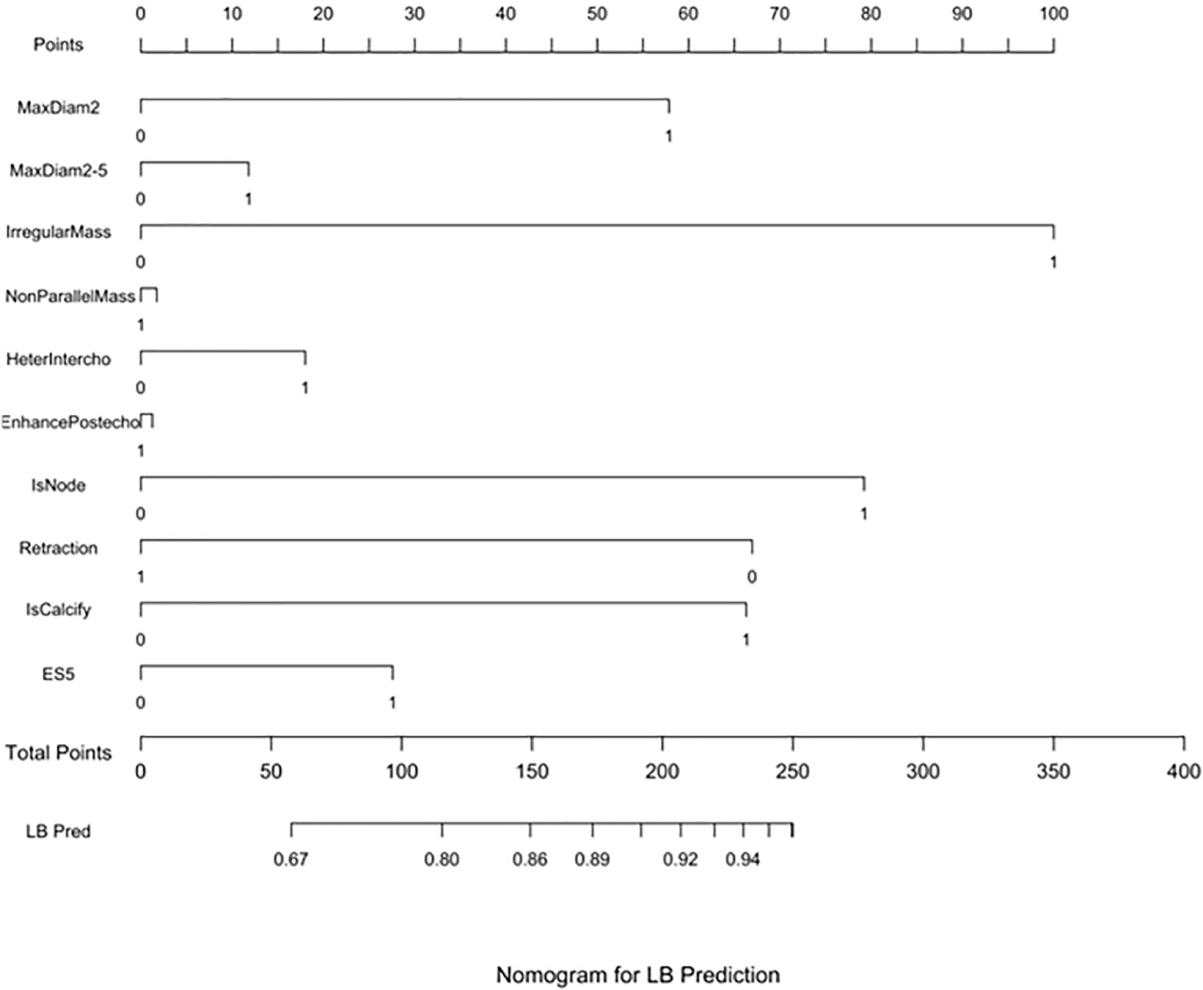
LB prediction nomogram. Nomograms were constructed based on the results of the multivariate logistic regression mode factors.

**Figure 2:**
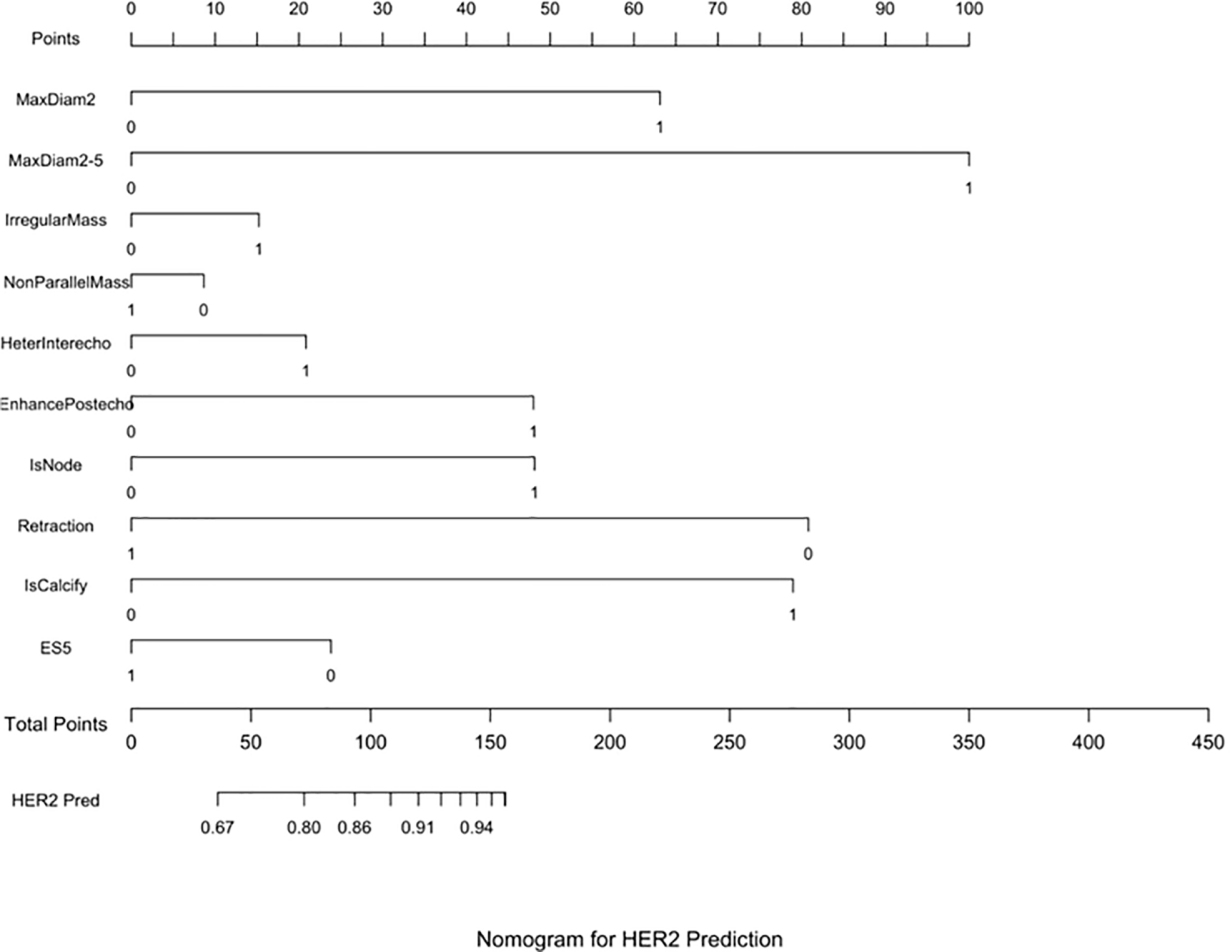
HER2 prediction nomogram. Nomograms were constructed based on the results of the multivariate logistic regression mode factors.

**Figure 3:**
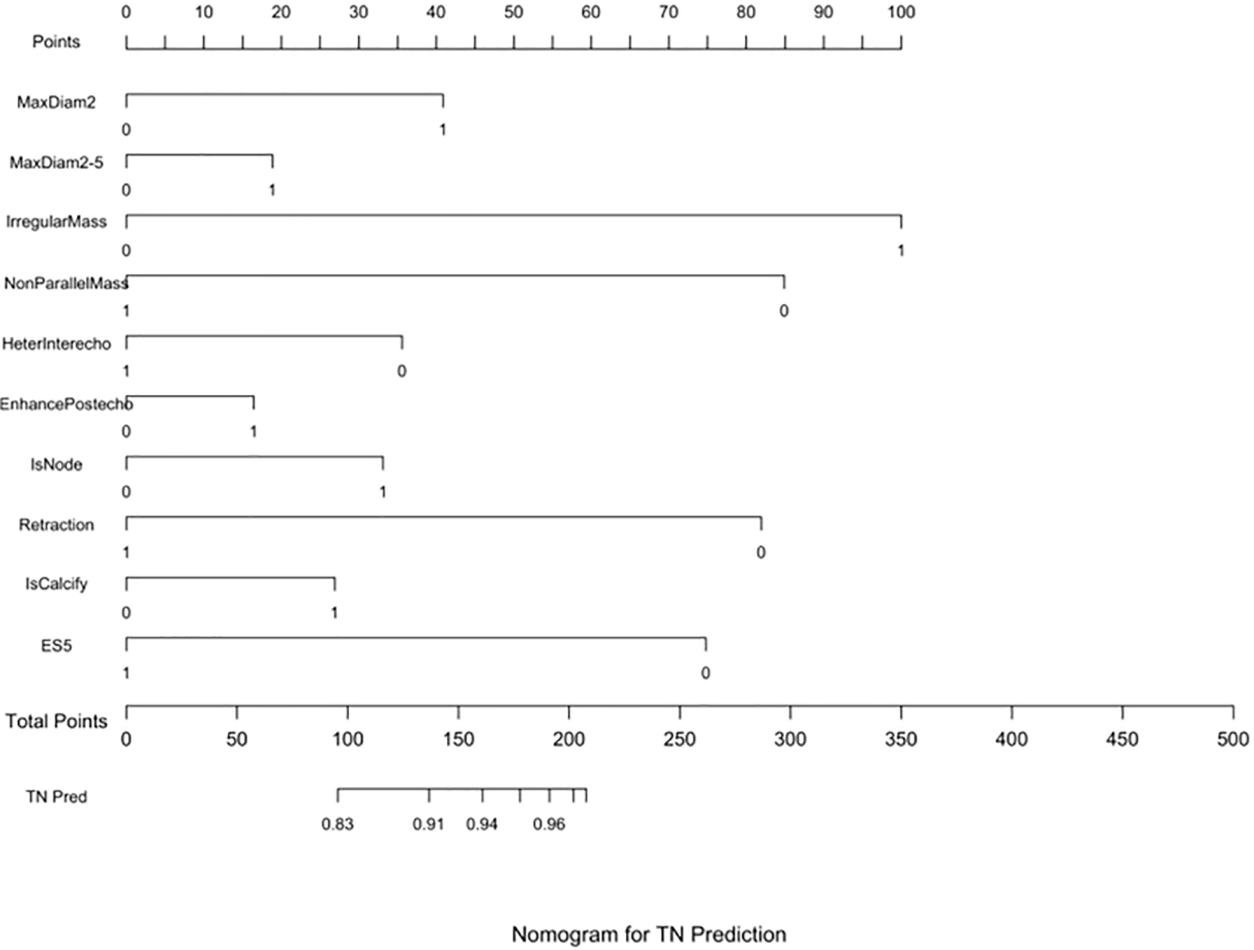
TN prediction nomogram. Nomograms were constructed based on the results of the multivariate logistic regression mode factors.

**Figure 4:**
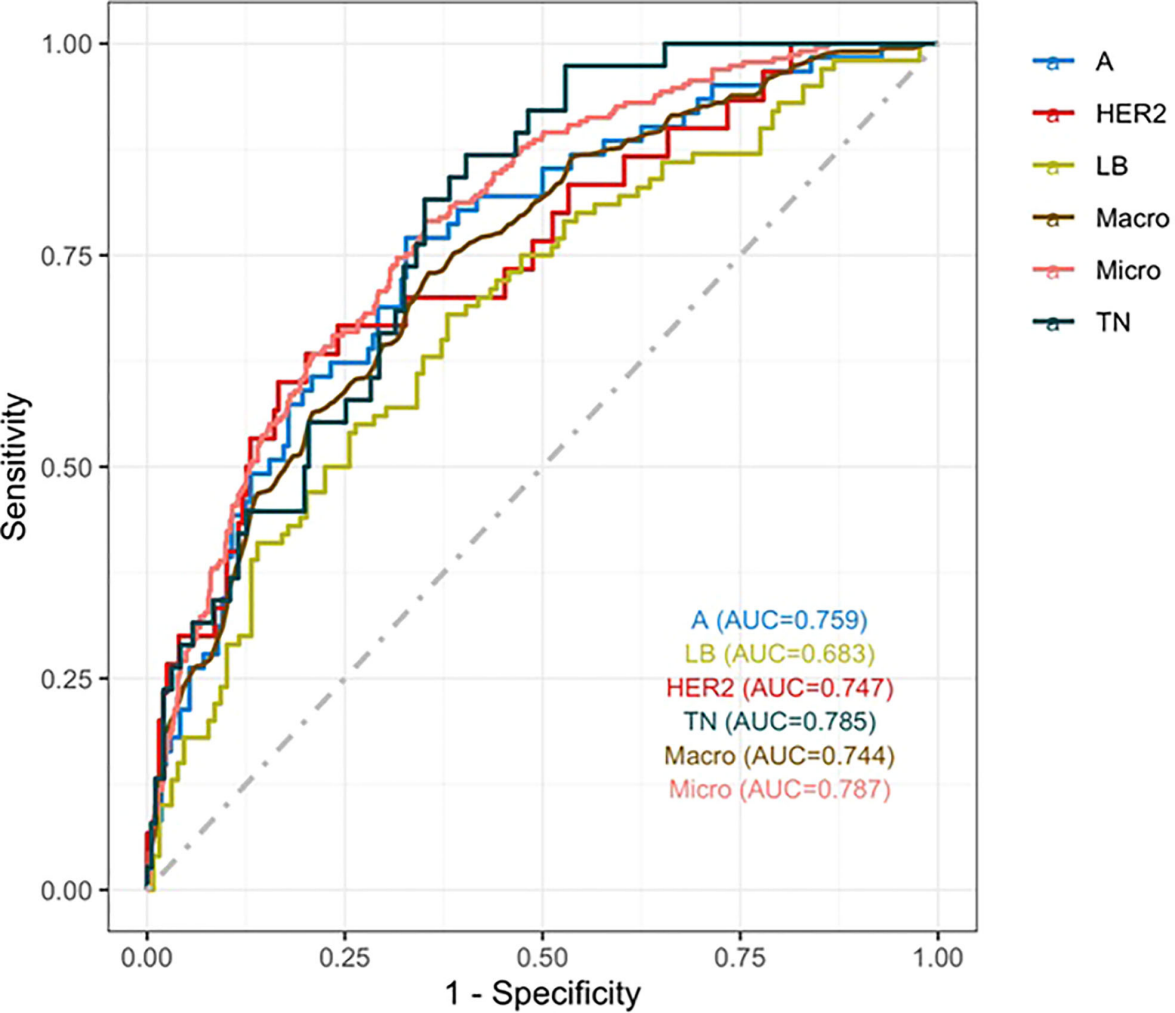
ROC curve. The macro- and micro-AUC were 0.744 and 0.787, respectively. The AUC was 0.759for predicting LA, 0.683 for predicting LB,0.747 for predicting HER2-positive, and 0.785 for predicting TN.

**Figure 5:**
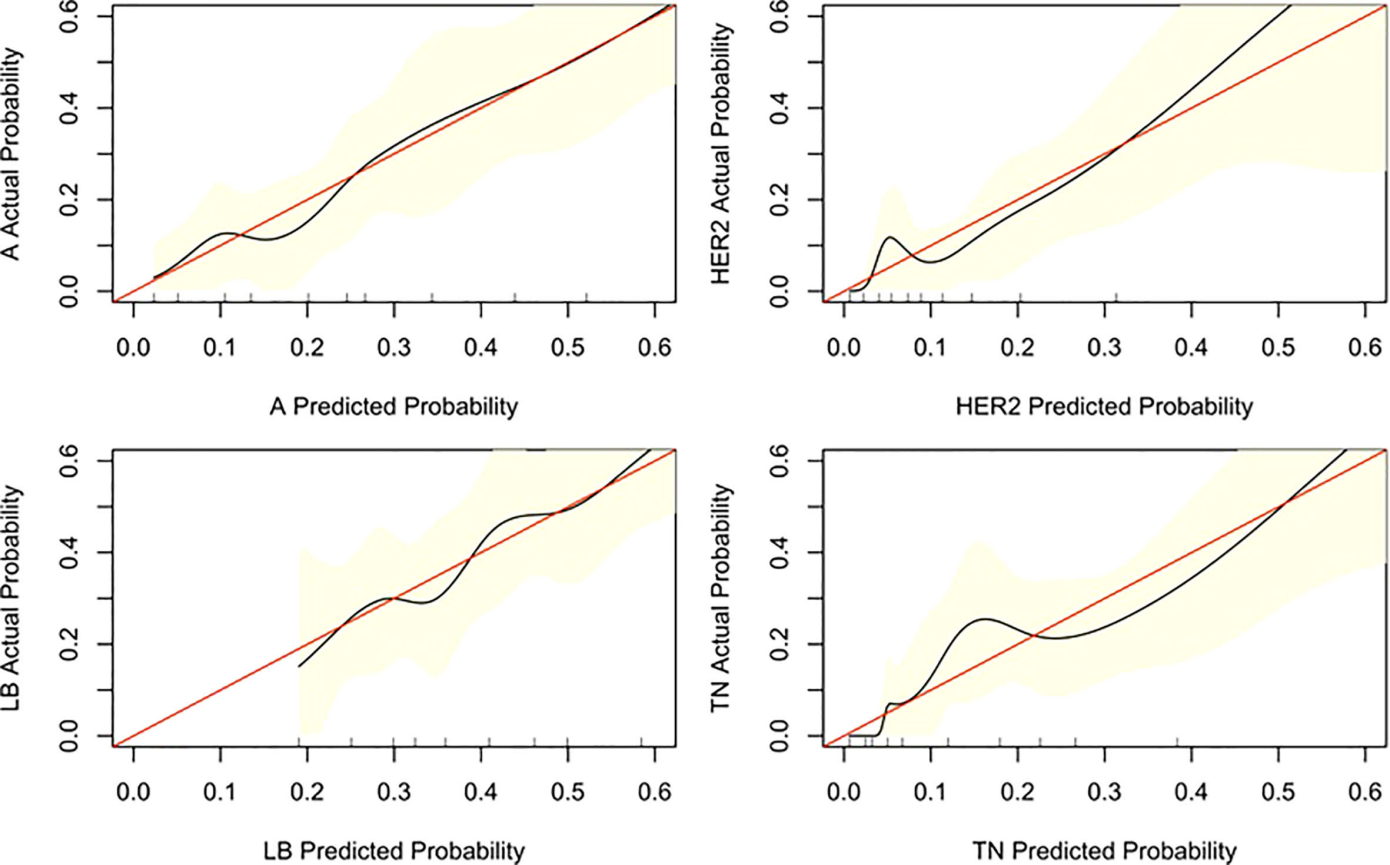
Calibration curve of the nomogram. The diagonal line indicates the ideal nomogram reference.

Three nomograms were created to predict the probabilities of the LB, HER2-positive, and TN subtypes. Each independent variable value corresponded to a point value on the top row, and the individual scores were added to yield the total score. The probability was then calculated based on the total score (15). The results are shown in **Figures 1**–**3**. Receiver operating characteristic (ROC) curves were established to assess the accuracy of the model (**Figure 4**). The macro- and micro-AUC were 0.744 and 0.787, respectively. The AUC was 0.759 for predicting LA, 0.683 for predicting LB,0.747 for predicting HER2-positive, and0.785 for predicting TN. Calibration curves were also constructed. The closer the calibration curve (black)is to the standard curve(red), the better the calibration capability of the model. The model had good calibration for the LA, HER2, and TN subtypes, as shown in **Figure 5**.

## Discussion

US is the most widely used auxiliary examination technique for the preoperative evaluation of breast cancer. However, certain errors may occur during the preoperative puncture, which can have adverse effects. Therefore, identifying the molecular classification of breast cancer is vital to guide the selection of an individualized clinical plan. Moreover, it is difficult to utilize the traditional classification for these treatment plans. Therefore, establishing a model that incorporates multiple factors to identify the molecular subtype can be used to predict the risk of mortality and facilitate the implementation of individualized treatment (18, 19). Since the ultrasonography features of different molecular types overlap, it is more appropriate to use a multivariate prediction model. The model in this study uses more parameters, thereby avoiding the limitations of single-parameter evaluation. The nomograms created here integrated relevant factors that affect molecular typing in a simple graphical manner, allowing clinicians and patients to understand the relationship between various factors and molecular typing. Given a set of known quantitative conditions, they can easily calculate the probability value of the corresponding outcome (20). Therefore, the realization of individualized classification prediction is in line with the pursuit of individualized treatment.

LA and LB cancers account for approximately 70% of breast cancer cases (21). Similar findings were observed in this cohort, where these subtypes accounted for 72.8% of cases (LA: 26.6% and LB:44.3%). Therefore, the distribution of molecular subtypes in this single-center study is consistent with those in other studies with larger samples.

ABVS is a 3D–US imaging system that overcomes the main shortcomings of HHUS, such as lack of standardization, non-repeatability, small field of view, and excessive time investment. Additionally, for women with dense breast tissue, ABVS can recognize calcification and the retraction phenomenon more due to its the unique coronal reconstruction. Furthermore, ABVS is more accurate than HHUS in assessing the extent of the disease, average lesion size (22), and largest diameter (23). Hence, it can better assess the ture scope of the disease. Recent studies have also shown that the imaging features acquired by ABVS are related to the molecular subtypes of breast cancer. In this study, there was a significant difference among the four molecular subtypes in the maximum diameter of the tumor. Patients with the LA subtype had tumors<2cm, whereas in patients with other subtypes, the largest diameter was 2–5cm. The HER2 molecular subtype had more calcification than the other subtypes. The retraction phenomenon was more likely to be seen in the LA subtype than in any other subtypes. The TN subtype did not display the retraction phenomenon orpost-acoustic enhancement. In the LA subtype, the body has more time to respond to cancer cells and form fibrosis,which leads to a contraction pattern (24, 25). Since ABVS has these advantages, it is moreusefulin evaluating breast cancer preoperatively.

A nomogram is a graphical calculation model that uses known predictive factors to calculate the numerical probability of a clinical event. Such prediction models are valuable. A multivariate logistic regression model we constructed *via* step wise analysis, Nomograms were subsequently developed based on the fitted multivariate logistic regression model.Tumor size, calcification, post-acoustic enhancement, and the retraction phenomenon were used to construct the nomogram model for distinguishing the molecular subtype of breast cancer. The scores of the influencing factors could show the individualized prediction results. The discriminative power of the nomogram was quantified using the AUC, exhibiting the accuracy of the test. Previous studies have shown that models with AUCs of 0.5–0.7 have low predictive value, models with AUCs of 0.7–0.85 have better predictive value, and models with AUCs of 0.85–0.95 have the best predictive value (26). The model in this study has AUCs of 0.68–0.78,indicating that the prediction model had a good degree of discrimination. The nomograms also contained information for clinical use; therefore, they might serve as tools to calculate the probabilities of the various molecular subtypes.

Recent studies have developed models to predict the molecular classification of breast cancer through radiomics or machine learning approaches using image segmentation (27, 28). These generally employ two-dimensional US modelling for a single molecular classification or deep learning magnetic resonance image modeling. Although these have been shown to be predictive, the feature extraction is complicated and has certain limitations. Compared with HHUS, ABVS allows the use of computer-aided design (29) and artificial intelligence technology to improve the diagnostic performance of deep machine learning due to its repeatability and image storage method. 3D-USmay potentially be useful in the field of artificial intelligence, however, this needs further verification. This study predicted the four molecular classifications using only 3D-US features. Nomograms we also used to visualize the predictive multinomial model of the molecular classifications.

The study has several limitations. First, this was a retrospective study performed at a single institution. A multicenter prospective study with a large sample size needs to be performed to validate this study’s results. Second, the sample sizes of the four molecular subtypes were unbalanced. As a result, the ROC curves of subtypes with fewer samples are unsatisfactory. Further studies should consider having balanced sample sizes.

## Conclusions

In conclusion, sonographic features such as calcification and posterior acoustic features were significantly associated with the breast cancer molecular subtypes. In addition, the presence of the retraction phenomenon was the most important predictor for the LA subtype. Nomogramsfor the prediction of the molecular subtypes were established based on the results of the multifactor analysis, The calibration and ROC curves showed that the model had good performance. Further multicenter studies will be useful for updating and validating these nomograms to improve the predictions of molecular subtypes.

## Data availability statement

The raw data supporting the conclusions of this article will be made available by the authors, without undue reservation.

## Ethics statement

The studies involving human participants were reviewed and approved by Hangzhou First people’s Hospital ethics committee. Written informed consent for participation was not required for this study in accordance with the national legislation and the institutional requirements.

## Author contributions

LB contributions to design of the work. YT and LY analysis of data for the work, XX and LL, drafting the work. LZ revising it critically for important intellectual content. All authors agree to be accountable for all aspects of the work in ensuring that questions related to the accuracy or integrity of any part of the work are appropriately investigated and resolved.

## Funding

The study was supported by The Construction Fund of Medical Key Disciplines of Hangzhou.

## Conflict of interest

The authors declare that the research was conducted in the absence of any commercial or financial relationships that could be construed as a potential conflict of interest.

## Publisher’s note

All claims expressed in this article are solely those of the authors and do not necessarily represent those of their affiliated organizations, or those of the publisher, the editors and the reviewers. Any product that may be evaluated in this article, or claim that may be made by its manufacturer, is not guaranteed or endorsed by the publisher.

## Abbreviations

3D-US, three-dimensional ultrasonography; ABVS, automated breast volume scanner; AUC, area under the curve; ER, estrogen receptor; HER2, human epidermal growth factor receptor 2; HHUS, hand-held ultrasonography; LA, luminal A; LB, luminal B; PR, progesterone receptor; ROC, receiver operating characteristic; TN, triple-negative.
